# High dose rate brachytherapy as monotherapy for localised prostate cancer: a hypofractionated two-implant approach in 351 consecutive patients

**DOI:** 10.1186/1748-717X-8-115

**Published:** 2013-05-08

**Authors:** Nikolaos Tselis, Ulf W Tunn, Georgios Chatzikonstantinou, Natasa Milickovic, Dimos Baltas, Markus Ratka, Nikolaos Zamboglou

**Affiliations:** 1 Department of Radiation Oncology, Klinikum Offenbach, Starkenburgring 66, 63069, Offenbach, Germany; 2 Department of Urology, Klinikum Offenbach, Starkenburgring 66, 63069, Offenbach, Germany; 3 Department of Medical Physics and Engineering, Klinikum Offenbach, Starkenburgring 66, 63069, Offenbach, Germany

**Keywords:** Prostate cancer, Brachytherapy, High dose rate, Iridium, Monotherapy

## Abstract

**Background:**

To report the clinical outcome of high dose rate brachytherapy as sole treatment for clinically localised prostate cancer.

**Methods:**

Between March 2004 and January 2008, a total of 351 consecutive patients with clinically localised prostate cancer were treated with transrectal ultrasound guided high dose rate brachytherapy. The prescribed dose was 38.0 Gy in four fractions (two implants of two fractions each of 9.5 Gy with an interval of 14 days between the implants) delivered to an intraoperative transrectal ultrasound real-time defined planning treatment volume. Biochemical failure was defined according to the Phoenix Consensus and toxicity evaluated using the Common Toxicity Criteria for Adverse Events version 3.

**Results:**

The median follow-up time was 59.3 months. The 36 and 60 month biochemical control and metastasis-free survival rates were respectively 98%, 94% and 99%, 98%. Toxicity was scored per event with 4.8% acute Grade 3 genitourinary and no acute Grade 3 gastrointestinal toxicity. Late Grade 3 genitourinary and gastrointestinal toxicity were respectively 3.4% and 1.4%. No instances of Grade 4 or greater acute or late adverse events were reported.

**Conclusions:**

Our results confirm high dose rate brachytherapy as safe and effective monotherapy for clinically organ-confined prostate cancer.

## Background

In patients with clinically localised prostate cancer, high dose rate (HDR) brachytherapy (BRT) has emerged as a safe and effective monotherapeutic modality [1-8]. It has shown PSA relapse-free survival rates which are comparable to those of patients treated with permanent low dose rate (LDR) BRT [9-11], external beam radiotherapy (EBRT) [12-14] or radical prostatectomy [15]. In the absence of randomised clinical trials of sufficient size, however, the optimal therapeutic strategy for organ-confined disease remains controversial and treatment assignment appears influenced by physician bias and patient preference. In addition, despite publications of mature results revealing superior outcomes for HDR monotherapy over a range of risk groups [1-3,8,16], a variety of dose schedules and of risk definitions make uniform recommendations concerning the optimal dose regime difficult. Therefore, the objective of the present study was to contribute to available experience by reporting our mature results of HDR BRT in 351 consecutive patients treated with a hypofractionated two-implant treatment regime.

## Methods

### Patient selection and characteristics

Since 2002, we have treated more than 900 patients with HDR monotherapy for clinically localised prostate cancer. During this period, three different protocols were implemented reflecting an evolution aiming to improve clinical workflow and patient comfort. From January 2002 to February 2004, 141 patients were treated with one implant of four fractions of 9.5 Gy. From March 2004 to January 2008, 351 patients received two implants, separated by two weeks, each of two fractions of 9.5 Gy. From February 2008 to the present, our HDR monotherapy regime consists of three implants, spaced by three weeks, each of a single fraction of 11.5 Gy. This paper analyses in detail the clinical outcome of our two-implant approach during the four-year time frame of 2004–2008 when we treated consecutively a cohort of 351 patients. The description of the other two protocols with limited comparative analysis of the outcomes of all three protocols is the subject of a different study [17].

All 351 patients had histologically proven disease and were staged according to the American Joint Committee on Cancer, 6^th^ edition, staging guidelines. Pre-treatment staging included digital rectal examination, transrectal ultrasound (TRUS) and, if clinically indicated, computed tomography (CT)/magnetic resonance imaging (MRI) and bone scintigraphy. The Memorial Sloan Kettering group definition was used to classify patients into risk groups [18].

Eligibility criteria were clinically and radiographically organ-confined disease with freedom from lower urinary tract symptoms requiring treatment. In the case of high-risk patients, treatment was performed in men who rejected prostatectomy or EBRT, or who were not considered suitable for prostatectomy or definitive dose-escalated EBRT. However, treatment assignment was in all cases at the discretion of the treating physician. Patients were considered ineligible for monotherapy in cases of nodal or other distant metastases, previous pelvic EBRT for another malignant diagnosis or previous open surgery of the prostate.

A total of 70 patients (19.9%) received androgen deprivation therapy (ADT), 44 (62.8%) of whom were high-risk, 19 (27.1%) intermediate-risk, and seven (10%) low-risk. Hormonal therapy was prescribed neoadjuvantly and continued concurrently with radiation and adjuvantly for an overall duration of median nine months (range, 3–14 months). The duration of ADT for the subgroups of low-risk, intermediate-risk, and high-risk patients was 4 months (range, 3–6 months), 6 months (range, 6–10 months) and 9 months (range, 9–14 months), respectively. Final decisions concerning hormonal treatment were made by the referring urologists. Patient and tumour characteristics are shown in Table 1.

**Table 1:** Patient and tumour characteristics (n=351)

**Characteristics**	
Median follow-up (months)	59.3 (16.5-82.6)
Gland volume at implant (cc)	
mean	41
median	39 (16–107)
Age at treatment (years)	
mean	67.7
median	68.4 (44.1-82.2)
Pre-treatment PSA (ng/ml)	
mean	7.1
median	6.4 (2.0-34.1)
	n (%)
Stage	
T1b-c	129 (36.7%)
T2a	113 (32.1%)
T2b	55 (15.6%)
T2c	53 (15.0%)
T3a	1 (0.2%)
Gleason score	
≤ 6	281 (80.0%)
7	62 (17.6%)
> 7	8 (2.2%)
Pre-treatment PSA (ng/ml)	
≤ 10	324 (92.3%)
11-20	23 (6.5%)
> 20	4 (1.1%)
Age at treatment (years)	
< 60	51 (14.5%)
60-69	169 (48.1%)
≥ 70	131 (37.3%)
Androgen deprivation therapy	70 (19.9%)
Risk group	
Low	196 (55.8%)
Intermediate	81 (23.0%)
High	74 (21.0%)

### Brachytherapy protocol

**Figure 1:**
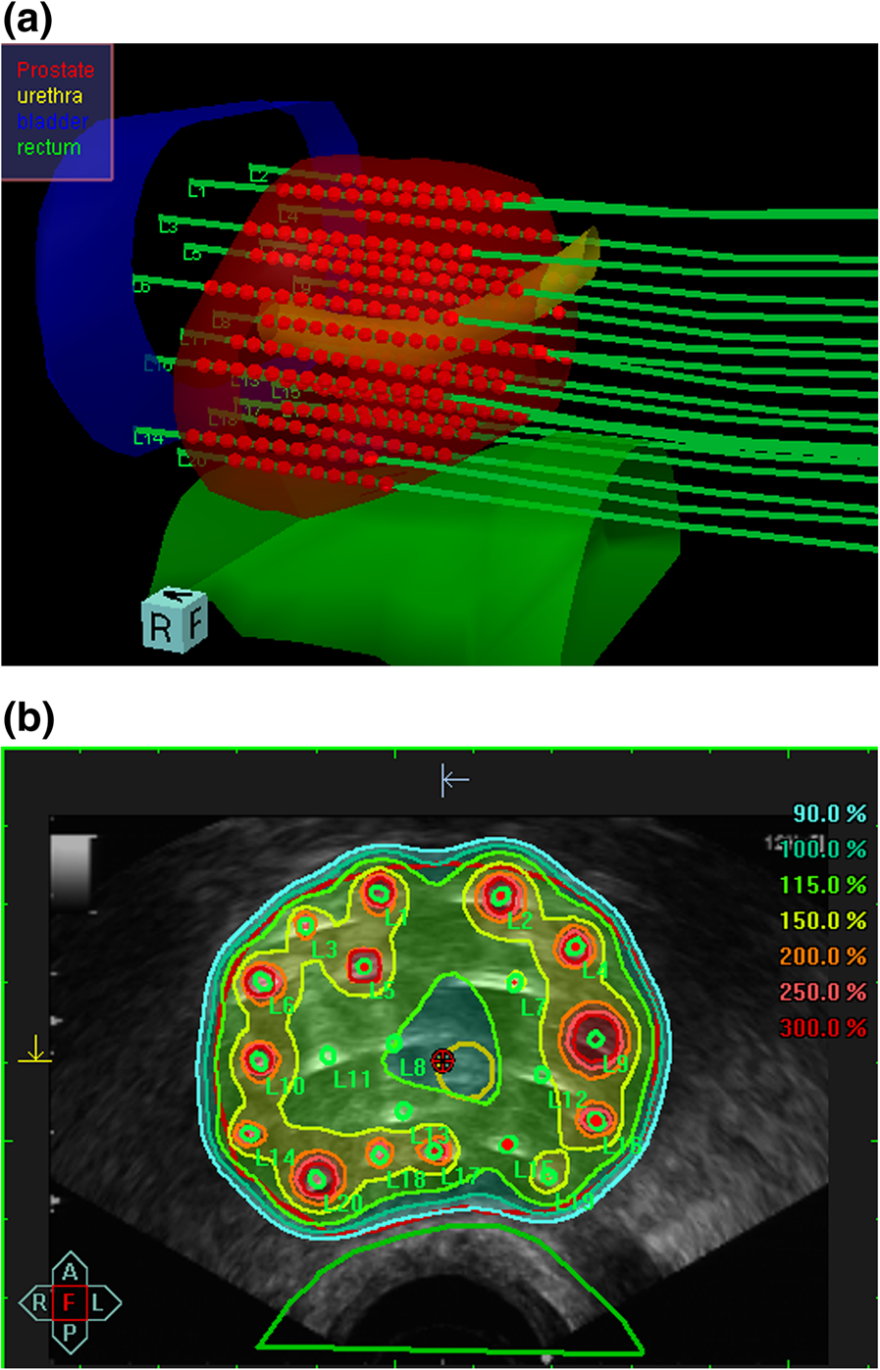
**Intraoperative real-time treatment planning. (a)** Three-dimensional reconstruction of the prostate, organs at risk (i.e., rectum, urethra, bladder), *in situ* needles and the intraprostatic source dwell positions as calculated using SWIFT for the final treatment plan. Contour definition for rectum extended 10 mm cranially from the prostatic base and 10 mm caudally from the prostatic apex. Urethra contouring encountered the intraprostatic urethra marked by the insertion of a three-way Foley catheter and extended by 5 mm caudally to include the apical urethra membrane. **(b)** Isodose distribution after anatomy-based dose optimisation. The isodose colour code convention is: dark red = 300% {isodose = 28.5 Gy}; orange = 200% {isodose =19.0 Gy}; yellow = 150% {isodose = 14.25 Gy}; turquoise = 100% {isodose = 9.5 Gy}. The 100% isodose was set to encompass the red delineated prostate capsule.

Transperineal implantation was performed under TRUS guidance using a continuous probe movement technique [19]. For preplanning, transversal ultrasound images of the prostate, urethra and anterior rectal wall were acquired in real-time and three-dimensional (3D) volumes reconstructed based on 1.0 mm image distance. The planning target volume (PTV) was defined as the entire prostate gland without margins. Based on the acquired 3D anatomy, the appropriate virtual needle positions were generated using the intraoperative treatment planning system SWIFT (Nucletron B.V., Veenendaal, The Netherlands). Afterwards the needle source dwell positions located within the prostate gland were activated and the radioactive source dwell times calculated by an anatomy-based optimisation algorithm. Dose volume histograms (DVHs) for prostate, urethra, and rectum were calculated for evaluation of the anatomy-based dose optimisation. As the preplanning dosimetry parameters fulfilled our dosimetric protocol (Table 2), TRUS guided implantation of steel needles (200 mm length, 1.9 mm diameter) was performed at the previously determined positions. After completion of implantation, a final 3D ultrasound data set was acquired (1.0 mm image acquisition) for intraoperative TRUS based real-time treatment planning. Prostate and organs at risk (OAR, i.e., rectum, urethra, bladder) contouring was checked and updated according to the new acquired image set. Real needle positions were reconstructed and dwell positions located within the prostate gland activated (Figure 1a). Using the anatomy-based optimisation tool of SWIFT the dwell times were automatically adjusted ensuring the resulted 3D dose distribution fulfilled our PTV coverage requirements in compliance with OAR dose constraints (Figure 1b). Evaluation of implant conformity was based on dose-volume parameters for the PTV and OARs. Dose specification was given as the mean dose on the PTV surface. The prescribed reference dose was 9.5 Gy delivered four times to a total dose of 38.0 Gy using two implants. The implants were spaced by two weeks, each delivering two treatment fractions with an interfraction interval of 6 hours. Dosimetry assessment parameters and OAR constraints are shown in Table 2.

**Table 2:** Dosimetry parameters of the brachytherapy protocol with total physical prescription dose (Gy) and normal tissue dose constraints (as percentages of the prescribed reference dose)

**Treatment scheme**	**PTV**	**D**_**10**_**/D **_**0.1 cm**_^**3**^	**D**_**10**_**/D **_**0.1 cm**_^**3**^	**D**_**10**_**/D **_**0.1 cm**_^**3**^	**D90**	**V100**	**V150**
		**Rectum**	**Bladder**	**Urethra**			
9.5 Gy x 4	38.0 Gy	≤ 75% / ≤ 80%	≤ 75% / ≤ 80%	≤ 115% / ≤ 120%	≥ 100%	≥ 90%	≤ 35%

PTV = planning target volume; D10-Rectum = dose delivered to 10% of the rectum; D10-Bladder = dose delivered to 10% of the bladder; D10-Urethra = dose delivered to 10% of the urethra; D _0.1 cm_^3^ = minimum dose to the most exposed 0.1 cm^3^ of the organ; D90 = dose delivered to 90% of the PTV ; V100, V150 = percentage of PTV receiving 100% and 150% of the prescription dose.

All implants were performed under spinal, or if indicated, general anaesthesia with the patient in the high lithotomy position using a perineal template. All treatments were performed using an ^192^Iridium HDR afterloading system (microSelectron-HDR, Nucletron). Written informed consent was obtained from all patients. In our series we had one patient with T3 disease, who was staged T3a. The PTV in this case was defined by the prostate capsule plus 5.0 mm in all directions (except for the posterior rectal margin). This was to cover extracapsular invasion as confirmed on pre-treatment pelvic MRI.

### Follow-up and statistical analysis

Initial follow-up was performed at six weeks after the last BRT fraction and then every three months for the first year, every six months for the second year and annually thereafter. Patient data was collected from a prospectively maintained database and by retrospective clinical chart review with data collection allowing also information acquisition from referring urologists. Toxicity analysis for gastrointestinal and genitourinary sites was performed using the National Cancer Institute Common Toxicity Criteria for Adverse Events, version 3. Follow-up for this analysis was completed during October 2010. During September and October 2010 all patients also received either at follow-up visits or by mail, a final questionnaire in order to assess the PSA status and to assess whether the last documented adverse events were persistent or not.

Acute toxicity was defined as symptoms that had completely resolved by six months after BRT. Adverse events that persisted or appeared beyond six months were considered late toxicity. Toxicity was scored per event and the highest value noted during follow-up was selected to calculate toxicity percentages. Biochemical relapse was defined according to the Phoenix definition (sustained post-treatment PSA value > nadir +2 ng/ml) [20]. Potency was defined as the ability to achieve an erection that was sufficient for intercourse. The estimated likelihood of events was calculated using the Kaplan-Meier method and comparisons made using the log-rank test. A two-sided P value ≤ 0.05 was considered statistically significant. For the multivariate analysis we used the Cox proportional hazards model.

## Results

### Clinical outcome

**Figure 2:**
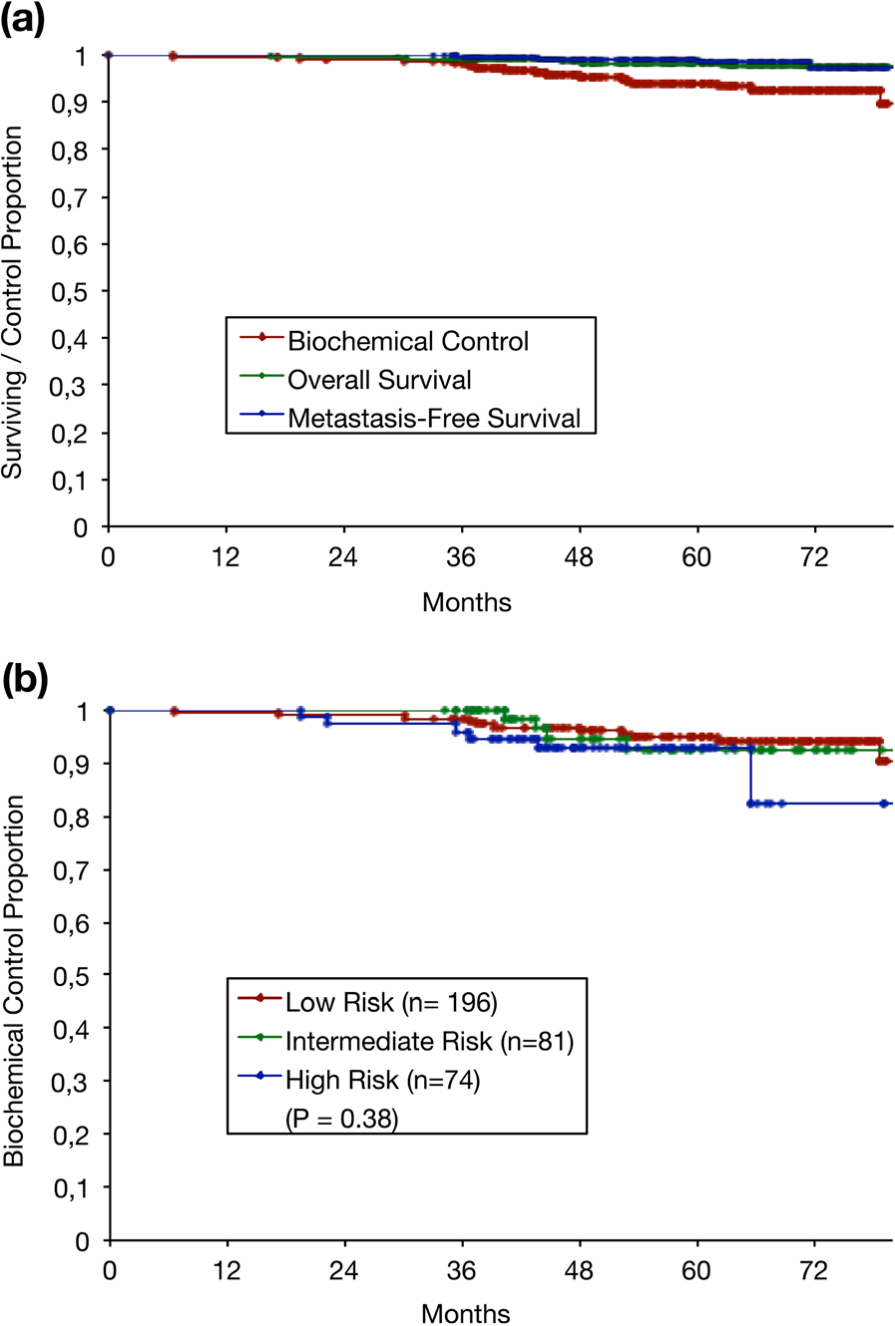
Kaplan-Meier actuarial analysis of all 351 patients for: (a) Biochemical control, overall survival and metastasis-free survival and (b) Biochemical control by risk group.

Of the 351 patients, 21 (5.9%) developed biochemical relapses with five (1.4%) developing distant metastatic disease. The estimated biochemical control (BC) was 98% at 36 months and 94% at 60 months. The estimated overall survival and metastasis-free survival were respectively 98%, 98% and 99%, 98% at 36 and 60 months. Survival and BC results are shown in Figure 2a.

The BC at 60 months according to risk group was 94%, 92%, and 92% for low-, intermediate- and high-risk patients respectively which did not achieve statistical significance, (P = 0.38, Figure 2b). The Kaplan-Meier estimates of BC at 60 months for the subgroup of high-risk patients who received ADT was 94% and 89% for patients without ADT, (P = 0.09). This was suggestive of a trend in favour of ADT, however it did not achieve statistical significance. The Kaplan-Meier estimates of BC at 60 months according to pre-treatment PSA were 93%, 100% and 100% for patients with an initial PSA of ≤ 10, 11–20 and > 20 ng/ml, (P = 0.43). There was hence no statistically significant difference. The BC according to clinical stage was 94%, 92% and 92% for patients staged ≤ T2a, T2b and ≥ T2c, (P = 0.37). Again, there was no statistically significant difference. The BC at 60 months according to Gleason score was 94%, 89% and 100% for patients with a Gleason score of ≤ 6, 7 and > 7, (P = 0.27) and also did not achieve statistical significance.

Characteristics used for multiple regression analyses to correlate with BC were clinical T stage (≤ T2a, T2b, ≥ T2c), Gleason Score (≤ 6, 7, > 7), pretreatment-PSA (≤ 10, 11–20, > 20 ng/ml) and ADT. The multivariate Cox regression analyses could not identify independent prognostic factors for biochemical failure.

The median follow-up was 59.3 months with 350, 340, 252 and 166 patients reaching the 24, 36, 48 and 60 month survival time points. Final follow-up questionnaires were returned by 344 (98%) patients during November and December 2010. Seven (2%) patients were not alive at the time of reporting. Follow-up details are given in Table 1.

### Toxicity

Table 3 shows the distribution of acute toxicity for the total group of 351 patients. Part of the data was presented at the 2007 German Society of Radiation Oncology meeting [21]. Table 4 shows the results for late morbidity. Seven (1.9%) patients developed late Grade 3 urinary retention requiring urethrotomy for urethral strictures. Another four (1.1%) patients developed late Grade 3 gastrointestinal toxicity, two of whom had endoscopically verified Grade 3 rectal mucositis. Treatment included endoscopic restoration of bowel continuity in one patient and a laser coagulation procedure for rectal bleeding in another patient. No Grade 4 or higher acute or late toxicities occurred.

**Table 3:** Acute toxicity results (n= 351)

	**Toxicity**	
Grade	Gastrointestinal	Genitourinary
Grade 5	0%	0%
Grade 4	0%	0%
Grade 3	0%	17 (4.8%)
Grade 2	6 (1.7%)	58 (16.5%)
Grade 1	55 (15.7%)	169 (48.1%)

**Table 4:** Late toxicity results (n = 351)

	**Grade**			
Toxicity	1	2	3	4
Genitourinary				
Frequency/Urgency	105(29.9%)	17 (4.8%)	2 (0.6%)	-
Dysuria	17 (4.8%)	4 (1.1%)	2 (0.6%)	0
Incontinence	30 (8.6%)	18 (5.1%)	1 (0.3%)	0
Retention	59 (16.8%)	19 (5.4%)	7 (2.0%)	0
Errectile Dysfunction	85 (24.2%)	55 (15.7%)	58 (16.5%)	-
Gastrointestinal (Rectum)				
Pain	7 (2.0%)	1 (0.3%)	1 (0.3%)	0
Mucositis/Necrosis	0	3 (0.8%)	4 (1.2%)	0
Diarrhea	0	0	0	0

Erectile dysfunction Grade 3, defined as a medically assisted erectile dysfunction inadequate for intercourse, was reported by 19 (5.4%) patients prior to BRT. Of the 332 patients who reported Grade 0–2 erectile dysfunction prior to treatment, 39 (11.1%) confirmed a decline to Grade 3 after BRT. In the final questionnaire 293 patients (83.4%) reported an erection adequate for intercourse achieved with or without the use of erectile aids.

## Discussion

Emerging radiobiological data indicates that prostate cancer has a low *a/β*-ratio of 1.2-3.0 Gy [22-24], suggesting that biological dose escalation can be achieved by hypofractionated treatment schemes. In that context, HDR BRT optimally exploits the radiobiological advantage of large fraction sizes while ensuring superior dose conformality. It enables 3D anatomy-based optimisation of the dose distribution by accurately controlling the radiation source positioning and modulating source dwell times. This permits excellent target coverage while at the same time minimizing dose to critical structures which can occur with LDR BRT due to source migration and tissue deformities [25].

The results from this study confirm HDR monotherapy as an excellent option for the definitive treatment of localised prostate cancer. The reported 94% BC at 60 months is in accordance with mature HDR monotherapy data from other institutions, suggesting high PSA relapse-free survival rates for all risk groups. In fact, consistent and reproducible five-year BC rates have been reported for patients with low-risk (85-97%), intermediate-risk (93-97%), and high-risk (79–88%) localised disease [1-8,16,26,27]. Even though direct comparisons are difficult, our results are consistent with the experiences of other groups using a similar two-implant approach. Mark et al. [1] reported an actuarial BC rate of 88% at eight years in 301 patients for all risk groups utilising two implants at three fractions of 7.5 Gy. Rogers et al. [8] reported a BC rate of 94% at five years in 284 intermediate-risk patients treated with two implants at three fractions of 6.5 Gy. Both institutions included clinical stages ≥ T2b with no exclusions for Gleason score or pre-treatment PSA in the series by Mark et al. [1]. These recently published data reflect that HDR monotherapy is applicable in intermediate as well as selected localised high-risk cases.

In our cohort, Gleason score, pre-treatment PSA, and clinical stage did not attain statistical significance using Kaplan-Meier actuarial estimates of freedom from biochemical failure. Rogers et al*.*[8], Hoskin et al*.*[3] and Yoshioka et al*.*[2] likewise failed to verify Gleason score and pre-treatment PSA or clinical stage as significant predictors of risk of biochemical failure. Hoskin et al. [3] reported on a group of 197 patients with a four-year BC rate of 87% in 86 high risk-cases. Those included clinical stages ≥ T3 in 21%, Gleason score ≥ 8 in 10%, and PSA > 20 in 25% of cases with 92% of high-risk patients receiving temporary ADT. In our study, the BC at 60 months for low-risk, intermediate-risk and high-risk patients was 94%, 92% and 92%, respectively. There was no statistically significant difference between these risk groups, indicating that all patients benefited equally. However, 60% of the high-risk patients received temporary ADT including all patients with PSA ≥ 20 ng/ml, 93% of patients with Gleason score ≥ 7b (4+3), and 52% of all cases staged > T2b. At this point, the potential advantage of short term androgen suppression for high-risk patients remains an issue of ongoing discussion as no corroborative evidence exclusive to this modality exists.

Gastrointestinal and genitourinary morbidity was low in our series with toxicity incidences across all scales consistent with those reported by other authors [1-3,5,8]. We encountered 3.4% late Grade 3 genitourinary toxicity with seven patients developing strictures requiring urethrotomy. Late Grade 3 rectal morbidity was 1.4% overall with one patient requiring endoscopic restoration of bowel continuity after rectal biopsy associated ulcer development. Additionally, in the final questionnaire 83.4% of patients reported erectile function, with or without the use of medical aids, suitable for intercourse. This rate of erectile preservation is consistent with data reported in recent publications on HDR monotherapy [8,26,27]. The consistently low level of severe acute and late toxicities in HDR BRT likely reflects the precision of 3D dosimetry, which can reduce uncertainties in dose distribution and thereby allow for better sparing of critical organs [28].

In accordance with the experience of other groups [8,28-30], neither larger gland size nor previous transurethral resection of the prostate (TURP) were absolute contraindications for treatment. The transperineal approach in high dorsal lithotomy position enables adequate implantation of volumes appreciably greater than 50 cc provided there is a sufficiently broad pelvic inlet and low pre-treatment urinary symptom scores. In the same way, implantation at six months after TURP was safely feasible given a sufficient amount of residual gland volume.

There are of course some limitations in our study. It encompassed 351 patients who were not treated within a multi-institutional framework but at a single tertiary-care center. The data was generated from a prospectively maintained database which was analysed by retrospective chart review. Our treatment modality has also limitations. Intraprostatic calcifications may impair TRUS imaging quality thereby limiting real-time US-based treatment planning and dose conformality. Therefore, the “technical eligibility” of each patient should be evaluated through a TRUS examination prior to treatment. Finally, the presence of seminal vesicle invasion or large prostate gland volumes extending beyond the bony pelvic inlet, also disqualify patients for monotherapy [28].

## Conclusions

The findings of the present analysis confirm monotherapeutic HDR BRT to be a safe and effective modality for clinically localised prostate cancer. Three-dimensional TRUS-based intraoperative real-time treatment planning provided accurate dosimetry with precise radiation delivery ensuring an acceptable side effect profile.

## Abbreviations

ADT: Androgen deprivation therapy; BC: Biochemical control; BRT: Brachytherapy; CT: Computed tomography; DVH: Dose volume histogram; EBRT: External beam radiotherapy; ED: Erectile dysfunction; HDR: High dose rate; IRT: Interstitial; LDR: Low dose rate; MRI: Magnetic resonance imaging; OAR: Organ at risk; OS: Overall survival; P: Level of statistical significance; PSA: Prostate specific antigen; PTV: Planning target volume; TRUS: Transrectal ultrasound; TURP: Transurethral resection of the prostate; US: Ultrasound; 3D: three-dimensional.

## Competing interests

None of the authors has any personal or institutional financial interest in drugs or materials described.

## Authors’ contributions

NT, UT and NZ performed study conception and design. NT, GC, MR and NM performed data collection, analysis and interpretation. DB assisted in data interpretation and manuscript writing. All authors read and approved the final manuscript.
